# Characterization of eukaryotic microbial diversity in hypersaline Lake Tyrrell, Australia

**DOI:** 10.3389/fmicb.2013.00115

**Published:** 2013-05-13

**Authors:** Karla B. Heidelberg, William C. Nelson, Johanna B. Holm, Nadine Eisenkolb, Karen Andrade, Joanne B. Emerson

**Affiliations:** ^1^Department of Biology, University of Southern CaliforniaLos Angeles, CA, USA; ^2^Department of Environmental Science, Policy, and Management, University of California, BerkeleyBerkeley, CA, USA; ^3^Department of Earth and Planetary Science, University of California, BerkeleyBerkeley, CA, USA

**Keywords:** microbial eukaryotes, diversity, hypersaline, saltern, *Dunaliella*, *Colpodella*, 18S rRNA

## Abstract

This study describes the community structure of the microbial eukaryotic community from hypersaline Lake Tyrrell, Australia, using near full length 18S rRNA sequences. Water samples were taken in both summer and winter over a 4-year period. The extent of eukaryotic diversity detected was low, with only 35 unique phylotypes using a 97% sequence similarity threshold. The water samples were dominated (91%) by a novel cluster of the Alveolate, Apicomplexa *Colpodella* spp., most closely related to *C. edax*. The Chlorophyte, *Dunaliella* spp. accounted for less than 35% of water column samples. However, the eukaryotic community entrained in a salt crust sample was vastly different and was dominated (83%) by the *Dunaliella* spp. The patterns described here represent the first observation of microbial eukaryotic dynamics in this system and provide a multiyear comparison of community composition by season. The lack of expected seasonal distribution in eukaryotic communities paired with abundant nanoflagellates suggests that grazing may significantly structure microbial eukaryotic communities in this system.

## Introduction

Hypersaline systems are distributed globally in the form of high salt lakes, salt ponds and solar marine salterns, where evaporative processes result in salt concentrations close to and exceeding saturation. Spurred on largely by advances in molecular biology, studies of microbial life in these systems are ideal because diversity is limited by the extreme conditions. In fact, several studies have characterized with great detail the dominant halophilic archaea and bacteria in hypersaline environments (e.g., Ventosa, 2006; Dyall-Smith et al., 2011; Ghai et al., 2011; Harris et al., 2012; Podell et al., 2013). The prokaryotic microbial and viral consortia in hypersaline Lake Tyrrell have been well described using deep metagenomic sequencing (Podell et al., 2013; Narasingarao et al., 2011; Fischer et al., 2012; Emerson et al., 2012, Submitted). These and other studies have placed microbes (used in this sense to include archaea, bacteria and viruses) as essential participants in most biogeochemical processes. However, studies of the diversity and activities of microbial eukaryotic (protistian) assemblages have lagged behind in this and other systems (Caron et al., 2009; Heidelberg et al., 2010). Consequently, the ecological importance that microbial eukaryotes have in food web dynamics in hypersaline systems is poorly understood (Pedrós-Alió et al., 2000; Elloumi et al., 2009). Further, a disproportionate percent of studies on microbial eukaryotes in hypersaline or saltern systems have focused on aspects related to halotolerant green alga, *Dunaliella* spp. (Oren, 2005; Ma et al., 2010; Ramos et al., 2011). The objectives of this study are to (1) characterize the eukaryotic microbial diversity in Lake Tyrrell in summer and winter over multiple years and (2) to compare water column microbial eukaryotes with those found in benthic salt crusts.

## Materials and methods

### Site description

**Figure 1:**
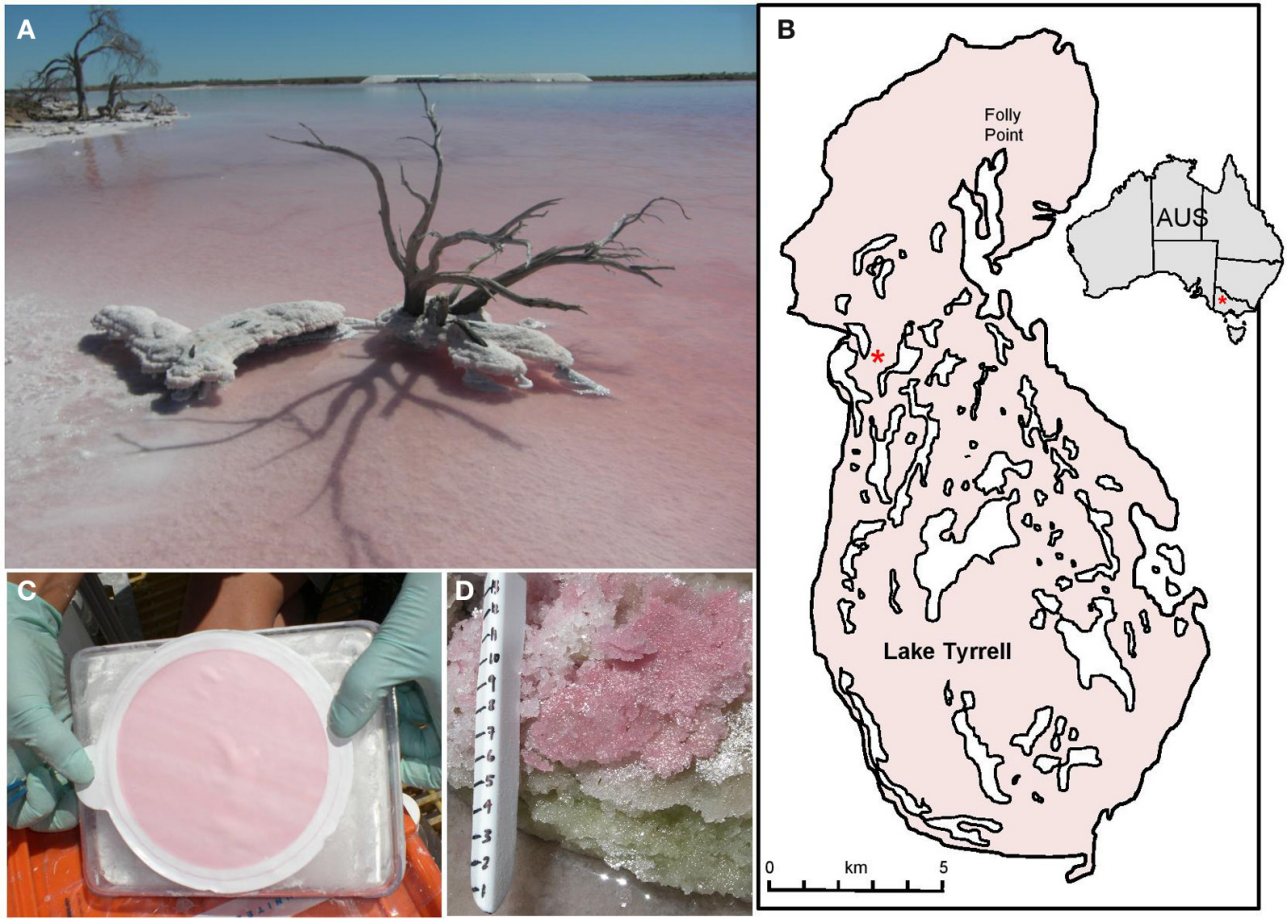
**(A)** Lake Tyrrell, Victoria Australia. (**B**) Map showing of the lake within Australia and the sampling site for this project (^*^). **(C)** 20–3.0 μm water fraction showing filter biomass. **(D)** Layered salt crust sampled for comparative purposes. Scale shows 1 cm increments (photo: J. Banfield).

Lake Tyrrell (LT) is an ephemeral, thalassohaline salt lake with an average surface area of 160 km^2^ that is located 320 km NW of Melbourne in semi-arid northwestern Victoria, Australia (Figure 1). The system experiences large seasonal fluctuations in temperature, salinity, oxygen, solar radiation, pH, and water levels. Water levels are controlled by the three sources of percolating brine groundwater, rainfall, and climate (Macumber, 1991, 1992). In winter, the lake contains ~50 cm water with a salt content ranging from 250 to 300 g L^−1^. In summer, water evaporates, leaving a halite crust up to 7 cm thick and residual brines with salt concentrations generally >330 g L^−1^. New brines emerge in springs along the eastern shore with salinities of ~100 g L^−1^, grading into the hypersaline waters toward the center of the lake. Less input comes from direct precipitation, localized runoff, and seasonal input from Lake Tyrrell Creek at the southern point of the lake.

### Sampling site characterization

Sample collection dates and site parameters are listed in Table 1. Average temperatures during sampling periods at the lake ranged from 21°C to 37°C in the dry, summer periods and 10–12°C in the wetter, winter season. The pH at the sample collection times varied between 6.93 and 7.30, but fluctuates greatly in other parts of the lake due to acidic groundwater and brine percolation. Total salinity was obtained by mass after evaporation of a known volume of water. Major ion composition was evaluated using established methods (Winge et al., 1979) at the Australia National University, Canberra, Australia. To evaluate nutrients, replicate 20 mL water samples were prefiltered through a 0.4 μm Nucleopore membrane filter and collected into acid washed polyethylene bottles. Samples were frozen on dry ice in the field and for transport. Samples remained frozen until analysis for nitrite/nitrate (Braman and Hendrix, 1989) and phosphorus (Huerta-Diaz et al., 2005). Nitrate+nitrite concentrations were variable and ranged between 1.40 and 10.3 μM in the summer and between 0.64 and 2.29 μM in the winter. The system shows continuously low phosphate concentrations between 0.01 and 0.05 μM. Additional chemical analysis of water samples indicated that the major ions in the water were Na^+^, Cl^−^, Mg^+2^, and SO^−2^_4_ (unpublished data, J. Brocks).

**Table 1:** **Samples collected in the central part of the lake between 2007 (S = summer) and 2010 (W = winter)**.

**Date (mm/dd/yy)**	**Sample ID**	**Water temp (°C)**	**Salinity (ppt)**	**TDS (wt/v%)**	**pH**	**NO**_**3**_ **+ NO**_**2**_ **(μM)**	**PO**_**4**_ **(μM)**
**SITE AH2 WATER SAMPLES**							
01/23/07 (S)	LT19	22	127	31.0	7.23	10.03	0.04
01/25/07 (S)	LT31	28	132	31.3	7.09		
08/07/08 (W)	LT35	12	101	28.0		1.96	0.05
08/11/08 (W)	LT41	12	109	27.5	7.21	2.10	0.01
08/12/08 (W)	LT42	11	114	30.1	7.30	2.29	0.04
01/03/09 (S)	LT50	21	161	35.0	6.97	2.05	0.03
01/07/09 (S)	LT62	27	155	34.5	6.93	1.40	0.05
01/06/10 (S)	AH2	37	153	34.0	7.22		
01/10/10 (S)	LT85	37	172	36.0	7.05		
**BENTHIC SALT CRUST SAMPLE**							
08/09/08 (W)	LT37 (crust)	10				0.64	0.04

TDS, total dissolved solids; ND, no data.

### Eukaryotic microbial sampling

Sampling for microbial eukaryotes took place at one site in the central region of the lake where a more permanent water body is found year around (35°19′ 09.6″ S, 142°47′ 59.7″ E; Figure 1). Surface water samples (0.3 m) were sequentially filtered through a 20 μm Nitex prefilter then 3.0, 0.8, and 0.1 μm 142 mm diam. Polyethersulfone filters (Pall Corporation) to obtain fractions enriched for a particular cell size (Narasingarao et al., 2011). Filters were placed in 50 mL centrifuge tubes with 10 mL DNA lysis buffer (100 μL TE buffer, 200 μL 1 M EDTA, 200 μL 0.5 M EGTA and 10 mL DI water). Halite crust samples (Figure 1D) were collected in 50 mL centrifuge tubes for analysis as a contrast to water column samples. All sampling tubes were frozen on dry ice or in liquid nitrogen for transport.

### Sequencing

Genomic DNA was extracted from ten samples using either the MoBio Power Soil DNA kit or a modified phenol extraction (Rusch et al., 2007). 18S rRNA genes were amplified from 10 to 70 ng of DNA from each sample using three universal eukaryotic primers EukA (5′-AACCTGGTTGATCCTGCCAGT-3′), EukB (5′-GATCCTTCTGCAGGTTCACCTAC-3′) (Medlin et al., 1988) and the internal primer 528F (5′-GCGGTAATTCCAGCTCCAA-3′) (Terrado et al., 2011) and by following the PCR protocol in the TOPO TA Cloning Kit for sequencing (Invitrogen). An ABI thermocycler (model 2720) was used with the following program: 95°C for 2 min, 35 cycles of 95°C for 30 s, 55°C for 30 s, and 72°C for 2 min and finally 72°C for 7 min. PCR products were separated on a 1.5% agarose gel, and the products of the expected size (~1900 bp) were excised and purified using the QIAquick gel purification kit (Qiagen) according to the manufacturer's protocol. PCR fragments were further cleaned and concentrated with a Clean and Concentrator-5 Kit (Zymo). Fragments were cloned into the pCR2.1-TOPO Vector using the TOPO TA cloning kit (Invitrogen) and transformed into TOP10-competent cells by electroporation (BTX ECM electroporator, Holliston, MA). Subsequently, the bacterial clones were plated and picked according to standard protocol. Sanger sequencing was performed on an ABI 377 machines at either at the Genome Center at Washington University (samples LT41 and LT42) or at the JCVI (all other samples).

### Sequence analysis

Raw Sanger sequences were trimmed using Phred at the default error probability cutoff of 0.05. Putative chimeras were identified in two steps. First, trimmed reads were screened against the full eukaryotic Silva database using UCHIME (Edgar et al., 2011). Sequences from the same clone for which the forward, reverse and internal reads all passed quality and chimera screens were then assembled using the Phred/Phrap/Consed programs (Ewing and Green, 1998; Ewing et al., 1998). Assembled contigs were rescreened for chimeras using Chimera Slayer (Haas et al., 2011). Less 0.2% of assembled clones were found to be chimeric.

Mothur, v1.26.0. (Schloss et al., 2009) was used for alignments and groupings for alpha diversity analysis. Assembled sequences were aligned against the SILVA database (Quast et al., 2013), screened and trimmed to columns 1109–42,675 of the SILVA alignment. Trimmed sequences were classified into operational taxonomic units (OTUs) using the average neighbor Mothur cluster algorithm. Groupings at 99%, 97%, and 95% nucleotide identity were examined to compare diversity between individual and groups of samples. Taxonomic assignments were made at 97% identity using the RDP naïve Bayesian classifier and the eukaryotic SILVA database (v.1.08) (Pruesse et al., 2007) with settings of kmer = 9 analysis and 100 iterations (Wang et al., 2007). Using this method, the relative abundance of groups within communities could be reliably estimated. Similarity of sample communities was examined using MDS plots generated using Log (x + 1) transformed abundance data and Plymouth Routines in Multivariate Ecological Research (PRIMER) software (Clarke and Gorley, 2006).

To further evaluate taxonomic relatedness of LT organisms, Geneious Pro software (v 6.0, Biomatters; http://www.geneious.com/) was used to generate ClustalW multiple alignments. Alignments were manually edited and imported into MEGA5 software (Tamura et al., 2011) for generation of maximum likelihood (ML) bootstrapped consensus trees based on the Hasegawa-Kishino-Yano model with five discrete Gamma distributions. Several versions of trees were initially constructed to identify representative LT sequences from larger groups of sequences that clustered together at high percent identity. While not quantitative, this allowed for the development of meaningful trees to evaluate relatedness of LT sequences to other groups.

### Light microscopy

In summer 2010, 10 water subsamples of 1 mL each were preserved in 1% paraformaldehyde and enumerated in a Sedgewick Rafter counting cell slide under 400× magnification using a compound Olympus, Model BX51 epifluorescent phase microscope (Woelkerling et al., 1976). Cell numbers were calculated from the average number of cells per field the field sample volume, specified by a grid on the bottom of the chamber.

## Results

### 18S rRNA gene clone libraries

After quality screening, our dataset contained a total of 2164 near full-length 18S rRNA gene sequences: 1392 from summer samples, 450 from winter samples and an additional 322 sequences from a benthic salt crust sample (Table 2). Sequences have been deposited in NCBI under accession numbers KC485974 to KC488158.

**Table 2:** **Summary of Lake Tyrrell near full length 18S rRNA Sanger sequences**.

**Date (mm/dd/yr)**	**ID**	**# Raw reads**	**% Good**	**Avg. read length**	**# Assem. (near full length)**	**Avg. assem. length (±SD)**	**Assem. used in analysis**
1/23/07 (S)	LT19	1152	86.9	732	253	1715.4 (15.9)	232
1/25/07 (S)	LT31	1152	81.5	783	237	1715.8 (11.1)	236
8/07/08 (W)	LT35	1152	91.8	708	261	1712.5 (12.8)	259
8/11/08 (W)	LT41	576	84.9	705	118	1631.0 (56.1)	108
8/11/08 (W)	LT42	576	83.7	872	91	1714.8 (34.7)	83
1/03/09 (S)	LT50	1152	87.4	667	194	1715.9 (10.6)	193
1/07/09 (S)	LT62	1152	94.4	735	233	1714.2 (18.0)	229
1/06/10 (S)	AH2	1152	91.8	767	290	1709.4 (38.1)	214
1/10/10 (S)	LT85	1152	89.1	805	290	1706.0 (24.4)	288
8/08/08 (W)	LT37 (crust)	1152	94.4	796	326	1709.3 (15.5)	322
Total			88.6	757.0	2226	1704	2164

Water samples were grouped by season and normalized to sequencing effort for several diversity estimates. Using Good's nonparametric coverage estimator (Esty, 1986) for grouped summer and winter samples, we found that our sampling depth captured 98% of water column diversity at 97% sequence similarity in both seasons for the water samples. Crust sampling effort was lower (1 sample); however, results from the Good's estimate suggested even with the lower sampling effort, we captured about 96% of the diversity within the crust sample. Both crust sample diversity and evenness were higher than the pooled summer and winter water samples, even though sampling effort was lower (Table 3).

**Table 3:** **OTU diversity statistics for 95%, 97%, and 99% similarities**.

**Identity (%)**	**# Seqs**	**OTUs**	**Goods coverage**	**Chao (CI)**	**Shannon (CI)**	**Shannon evenness**
**SUMMER**						
99	450	52	0.900	382 (170–974)	1.378 (1.20–1.55)	0.349
97	450	17	0.976	45 (24–131)	0.456 (0.33–0.58)	0.161
95	450	9	0.984	30 (14–95)	0.249 (0.16–0.34)	0.113
**WINTER**						
99	450	60	0.887	378.8 (182.1–891.9)	1.46 (1.27–1.65)	0.357
97	450	16	0.980	25 (18–56)	0.44 (0.32–0.56)	0.156
95	450	8	0.990	13 (9–40)	0.24 (0.15–0.32)	0.114
**WINTER CRUST**						
99	323	55	0.860	303 (148–712)	1.77 (1.55–1.99)	0.442
97	323	20	0.960	46 (27–119)	0.94 (0.78–1.11)	0.316
95	323	16	0.900	34 (20–91)	0.64 (0.49–0.79)	0.230

CI, confidence interval. Summer and winter water samples normalized to the lowest sample effort (n = 450). The crust sample (LT37) is included for comparison but has lower sequencing effort than the grouped summer and winter samples.

**Figure 2:**
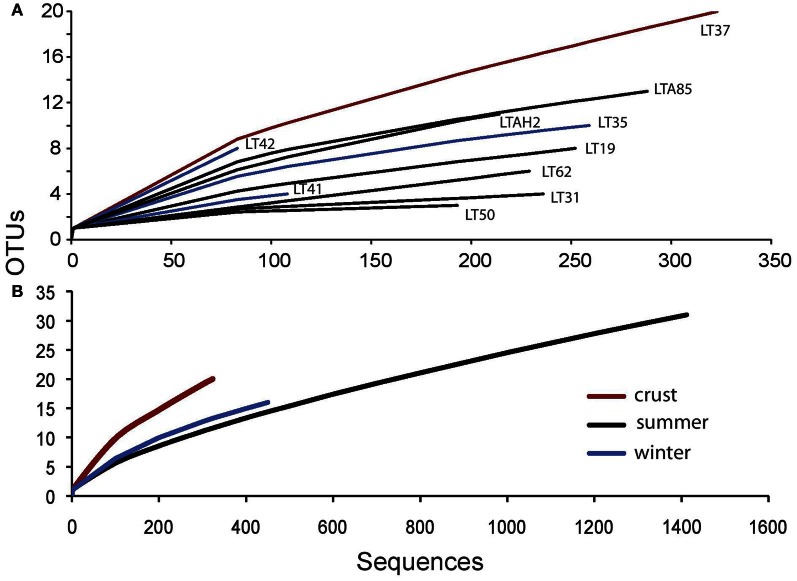
**Microbial eukaryote sample rarefaction analysis at 97% similarity. (A)** individual sample curves and **(B)** grouped sample curves for summer (*n* = 6), winter (*n* = 3), and crust (*n* = 1).

Sample-based rarefaction analysis (species accumulation curves) was used to show total species richness as a function of sequencing effort. Individual sample analysis and pooled summer and winter water samples vs. the halite crust sample also indicate the higher diversity in the crust sample (Figure 2).

### Community composition determined by clone libraries

**Figure 3:**
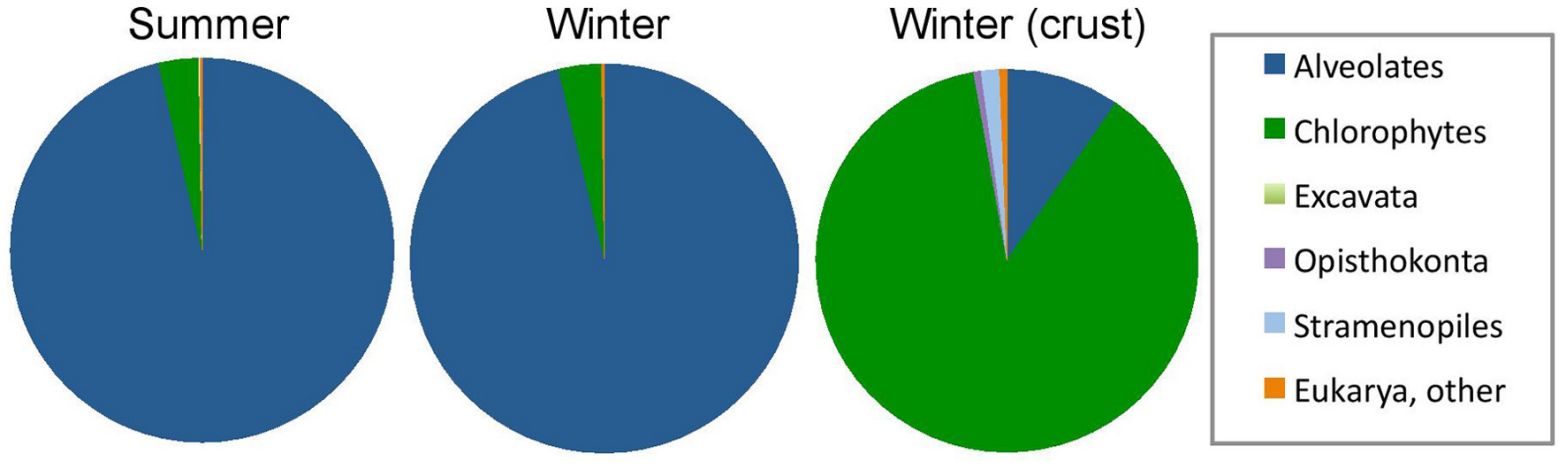
**Relative abundance of 18S rRNA sequences from summer samples (*n* = 6 samples; 1392 sequences) and winter samples (*n* = 3 samples; 450 sequences) and a benthic halite crust sample (crust) (*n* = 1; 322 sequences).** Relative taxonomic abundance data by sample is provided in Table 4.

Taxonomic analysis of the community 18S rRNA sequences provided better insight into community composition. Community membership in each sample was characterized by Bayesian inference at 97% similarity cutoff as described in the methods (Table 4). Detailed Bayesian classification data for each unique sequence is presented in Table S1. Both summer and winter water samples were dominated by Alveolates (96.3% of sequences recovered in summer and 96.2% in winter). Of these, 91.0% and 91.1% were closely related to *Colpodella* spp. in summer and winter water samples, respectively. The Chlorophyte, *Dunaliella* spp. accounted for 3.4% of sequences recovered in summer and 2.4% in winter. Sequences from other groups together made up <3% of the community (Figure 3; Table 4). Conversely, analysis of a representative winter halite crust sample was 91.1% *Dunaliella* and 7.5% other groups (Figure 3). Several additional groups were also seen in the halite crust, which were not seen in the water samples (Table 4).

**Table 4:** **Relative percent distribution of 18S rRNA sequences into taxonomic groups for each sample using a 97% cutoff Bayesian classification (S = summer; W = winter)**.

	**LT19 (S)**	**LT31 (S)**	**LT35 (W)**	**LT41 (W)**	**LT42 (W)**	**LT50 (S)**	**LT62 (S)**	**AH2 (S)**	**LT85 (S)**	**Crust LT37**
**CHROMALVEOLATA**										
Apicomplexa, *Colpodella* spp.	92.7	94.9	90.0	93.5	91.6	89.1	98.3	84.1	86.8	5.9
Apicomplexa, other	0.0	0.0	0.8	0.0	0.0	0.5	0.0	0.9	0.0	0.0
Ciliophora, Heterotrichea	0.4	0.0	0.0	0.0	0.0	0.0	0.4	0.0	1.0	1.2
Ciliophora, Plagiopylea	0.0	0.0	0.0	0.0	0.0	0.0	0.0	0.0	0.7	0.0
Ciliophora, Stichotrichia	0.0	0.0	0.0	0.0	1.2	0.0	0.0	0.0	0.0	0.0
Alveolata, other	4.3	0.0	7.7	0.0	0.0	0.0	0.4	14.5	8.0	2.5
**CHLOROPHYTA**										
Chlamydomonadales, *Dunaliella*	2.2	4.7	1.5	4.6	2.4	10.4	0.4	0.5	3.1	82.9
Chlamydomonadales, *Halosarcinochlamys*	0.0	0.0	0.0	1.9	2.4	0.0	0.0	0.0	0.0	2.8
Chlamydomonadales, other	0.0	0.0	0.0	0.0	1.2	0.0	0.0	0.0	0.0	1.9
**EXCAVATA**										
Discoba, Heterolobosea, *Vahlkampfiidea*	0.4	0.0	0.0	0.0	0.0	0.0	0.0	0.0	0.0	0.0
**OPISTHOKONTA**										
Choanoflagellida	0.0	0.0	0.0	0.0	0.0	0.0	0.0	0.0	0.0	0.3
Mesomycetozoa, Ichthyophonida	0.0	0.0	0.0	0.0	0.0	0.0	0.0	0.0	0.0	0.3
Fungi, Basidiomycota, *Malassezia*	0.0	0.0	0.0	0.0	0.0	0.0	0.0	0.0	0.3	0.0
**STRAMENOPILES**										
Bicoecea, *Caecitellus*	0.0	0.0	0.0	0.0	0.0	0.0	0.0	0.0	0.0	0.9
Bacillariophyta	0.0	0.0	0.0	0.0	0.0	0.0	0.0	0.0	0.0	0.6
**Unclassified, Eukarya**	0.0	0.4	0.0	0.0	1.2	0.0	0.4	0.0	0.0	0.6
Number sequences	232	236	259	108	83	193	229	214	288	322

**Figure 4:**
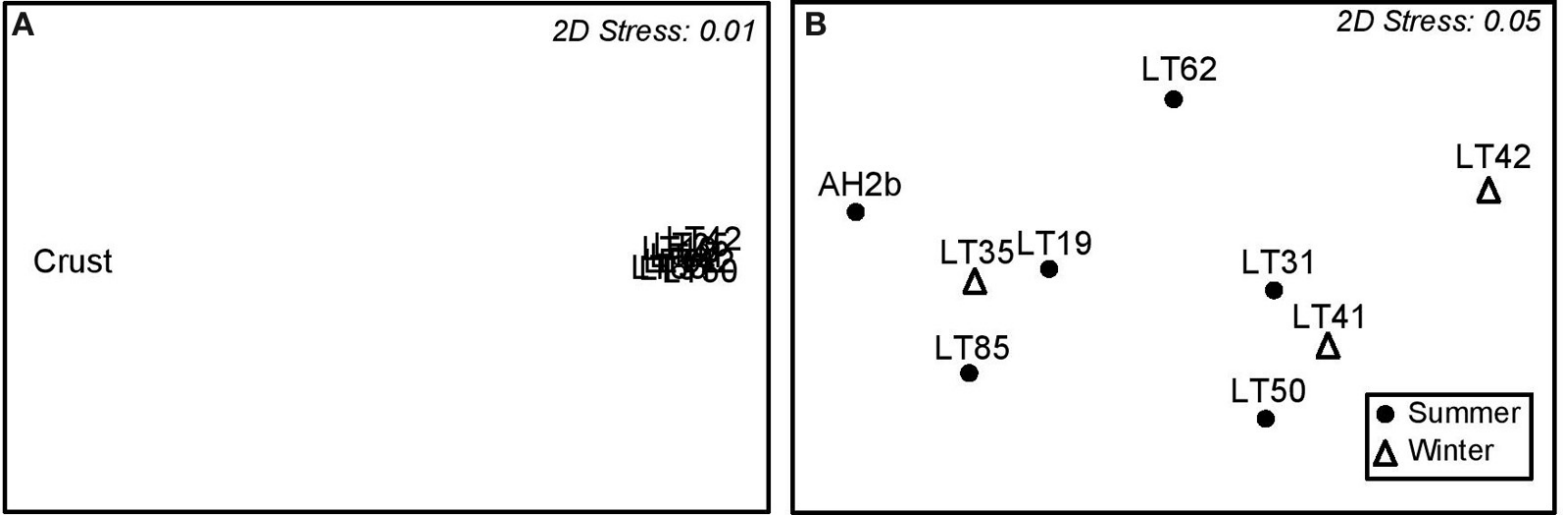
**S17 Bray-Curtis similarity multidimensional scaling (MDS) plots showing sample similarity. (A)** Clustering of all samples. **(B)** Clustering of only water samples.

Individual sample communities were compared using multidimensional scaling (MDS) analysis to provide an analysis of the pattern of proximities (i.e., similarities or distances) among samples. When all samples were compared, the benthic crust sample clustered well away from water samples, with a 2D stress factor of 0.01 (Figure 4A). However when the crust sample was removed, summer and winter water samples had no unique pattern of community clustering (2D Stress 0.05) (Figure 4B), despite physical parameters being variable in temperature (summer 28.67 ± 7.00; winter 11.67 ± 0.58°C) and salinity (summer 33.63 ± 2.04; winter 28.53 ±1.38 wt/v%) (Table 1).

### Phylogeny of 18S rRNA sequences

We generated ML trees to evaluate taxonomic positional of our sequences within specific groups. Given the number of sequences in the dataset, multiple iterations of trees were used to identify a range of representative sequences to compare to sequences in public databases. In many cases, sequences in trees represent additional sequences that are not shown. A full listing of taxonomic assignments using the RDP naïve Bayesian classifier and the eukaryotic SILVA database is in Supplementary Table 1.

#### Colpodella spp.

**Figure 5:**
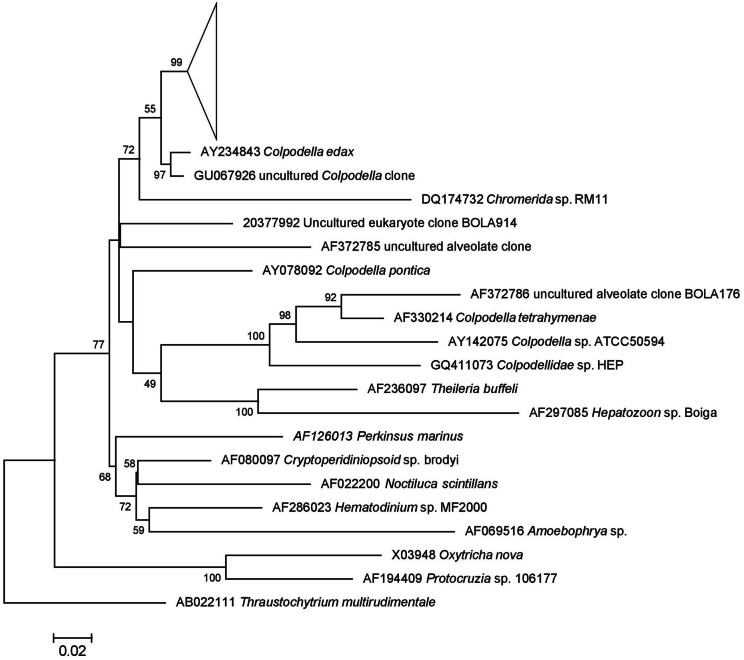
**18S rRNA ML tree of *Colpodella* (Apicomplexa) tree with 100 iterated bootstraps.** The tree was inferred from an alignment of 89 nucleotide sequences over 1678 bp based using the Hasegawa-Kishino-Yano model in MEGA5. All representative Lake Tyrrell sequences (*n* = 69) are found within the collapsed node. The scale bar represents the number of substitutions per site. The tree is rooted with *Thraustochytrium multiudimentale*.

**Figure 6:**
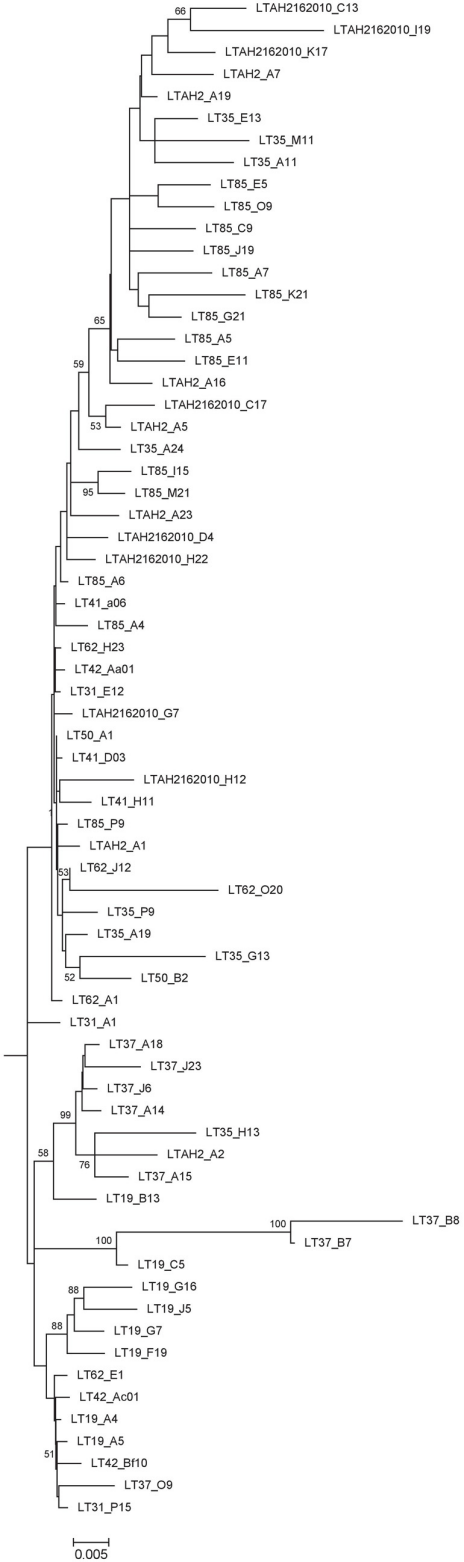
**Expansion of 69 Lake Tyrrell *Colpodella* sequences from Figure 5**.

A preliminary tree of the 1695 Lake Tyrrell colpodellid sequences was used to identify 69 representative sequences for further analysis. These sequences were realigned with reference *Colpodella* spp. and closely related apicomplexan sequences for the construction of an ML tree. Our sequences formed a discrete node with significant bootstrap support that was closest to a *C. edax* reference sequence (89.2–96.8% identity) (Figure 5). Within the node, sequence identities were between 89.1 and 99.9% similar (Figure 6).

#### Dunaliella spp.

**Figure 7:**
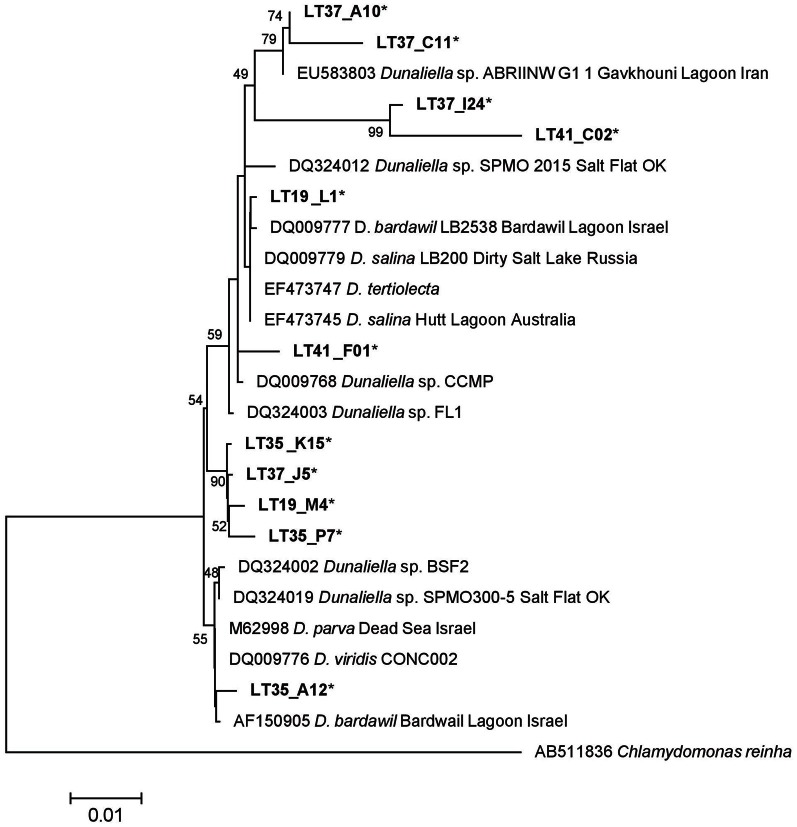
18S rRNA ML of *Dunaliella* sequences based on a 1496 bp alignment, including 11 representative LT sequences (^*^) with 13 *Dunaliella* public reference sequences. The tree is drawn to scale, with branch lengths measured in the number of substitutions per site using the Hasegawa-Kishino-Yano model and MEGA5 as described in the methods. Bootstrap values are shown as the percentages of 100 trees inferred in the analysis. The tree was rooted with *Chlamydomonas reinha*.

*Dunaliella*-related sequences (*n* = 325) were narrowed to 11 representative sequences and realigned with 13 reference strains. Many *Dunaliella* strains harbor introns in their 18S rRNA genes (Olmos et al., 2000; Hejazi et al., 2010). Intron regions, if present, were removed from both our sequences and reference sequences for the phylogenetic analysis. Unlike *Colpodella* (Figures 5 and 6), LT *Dunaliella* sequences were more diverse. Representative sequences aligned with a variety of described *Dunaliella* species found in other systems. There was one novel subclade with good support, but evolutionary differences between environmental clones and reference sequences were quite small. No sequence had >7% divergence from known species (Figure 7). Representative clones from halite crust samples aligned well with those from water column samples, indicating that the populations are not separate.

#### Ciliophora sequences

**Figure 8:**
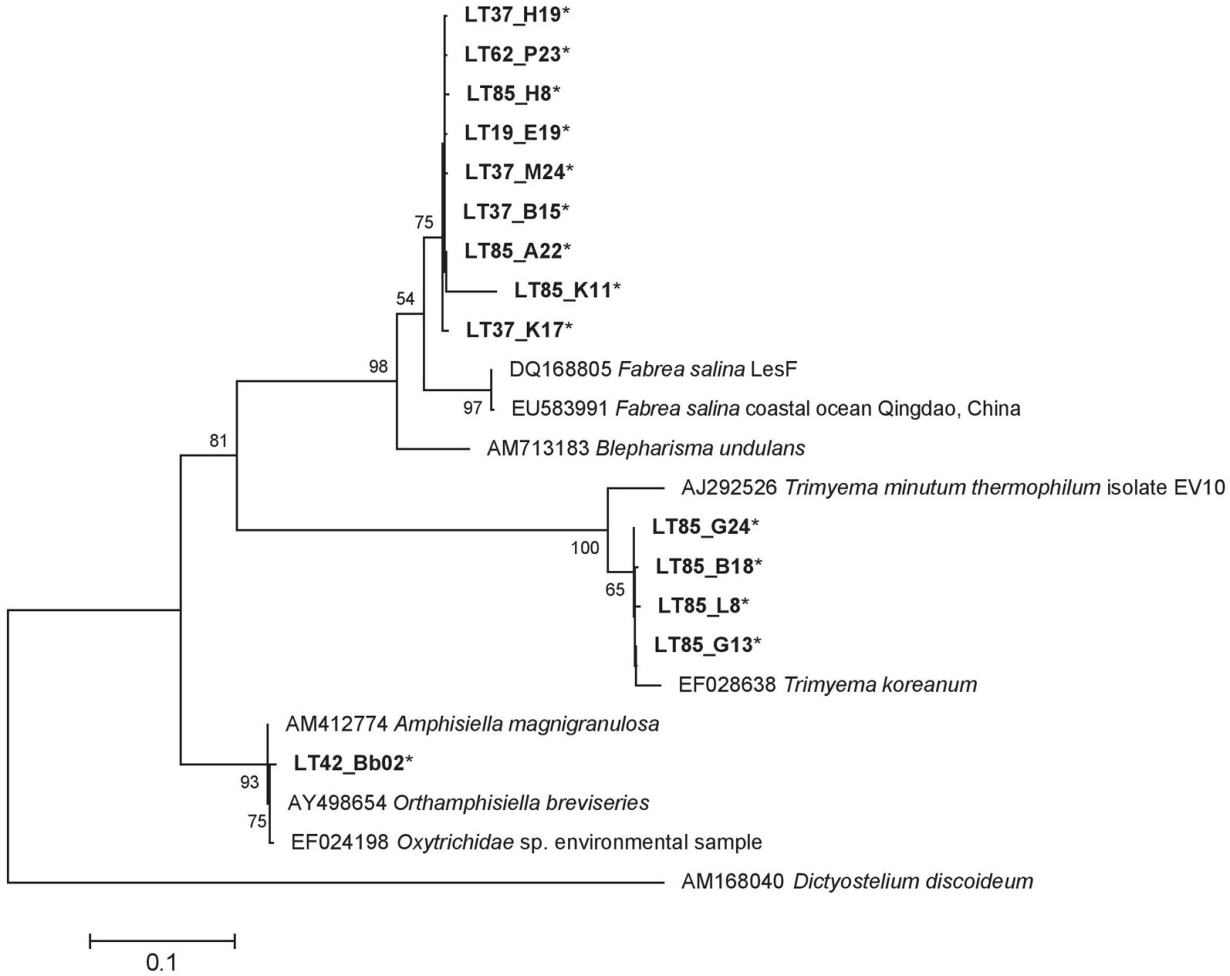
**18S rRNA ML tree of Ciliophora sequences generated from 23 sequences and a 1390 bp alignment.** There were 14 representative Lake Tyrell sequences (^*^) and 8 reference sequences. The tree is drawn to scale, with branch lengths measured in the number of substitutions per site using the Hasegawa-Kishino-Yano model and MEGA5 as described in the methods. Bootstrap values are shown as the percentages of 100 trees inferred in the analysis. The tree was rooted with *Dictyostelium discoideum*.

Our samples included 14 ciliate-related sequences associated with three major groups (Table 4). Of particular note, nine sequences formed a well-supported novel Heterotrichea subclade most closely related to *Fabrea salina*. These sequences came from both summer and winter samples and both water column and crust samples. Four sequences, all from sample LT85, clustered closely to another hypersaline Plagiopylea ciliate, *Trimyema koreanum*. The other sequence clustered most closely with a stichotrich ciliate, *Orthamphisiella breviseries* (Figure 8).

#### Stramenopile sequences

**Figure 9:**
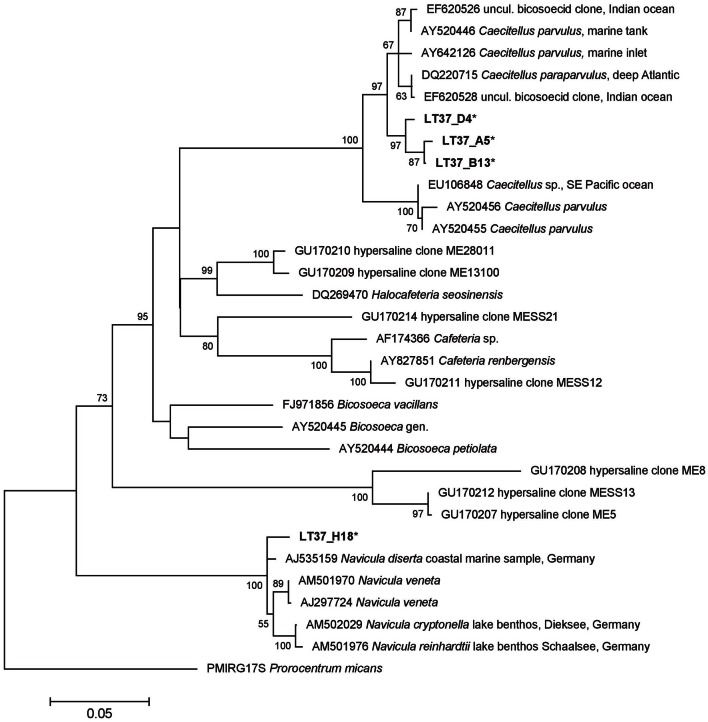
**18S rRNA ML tree of stramenopiles from 31 sequences and a 1519 bp alignment.** The tree was generated using a Hasegawa-Kishino-Yano model and MEGA5 as described in the methods. There were 4 Lake Tyrrell sequences (^*^). The tree is drawn to scale, with branch lengths measured in the number of substitutions per site. Bootstrap values are shown as the percentages of 100 trees inferred in the analysis, and the tree was rooted with the dinoflagellate, *Prorocentrum micans*.

No stramenopile sequences were found in water samples, but four were found in the halite crust sample (Table 4). Three of these sequences (LT37_D4, LT37_A5, LT37_B13) formed an independent node deeply within the ancestral Bicoecea, a phagotrophic heterotrophic nanoflagellate group (Figure 9). Our sequences formed an independent group within the *Caecitellus* genera, which are raptorial gliders commonly found in a variety of marine sediments and commonly thought to be a sister group to the Cafeteriaceae (Cavalier-Smith and Chao, 2006) (Figure 9). The most closely related public sequences to our samples were from marine environments, not hypersaline systems, which supports the notion that that this is a highly adaptive and diverse group. However the distinct grouping of our sequences also suggests that they may represent a distinct assemblage of halotolerant *C. parvulus*. Distinct halotolerant Bicosoecida groupings have been found for other stramenopile groups (Park and Simpson, 2010).

**Figure 10:**
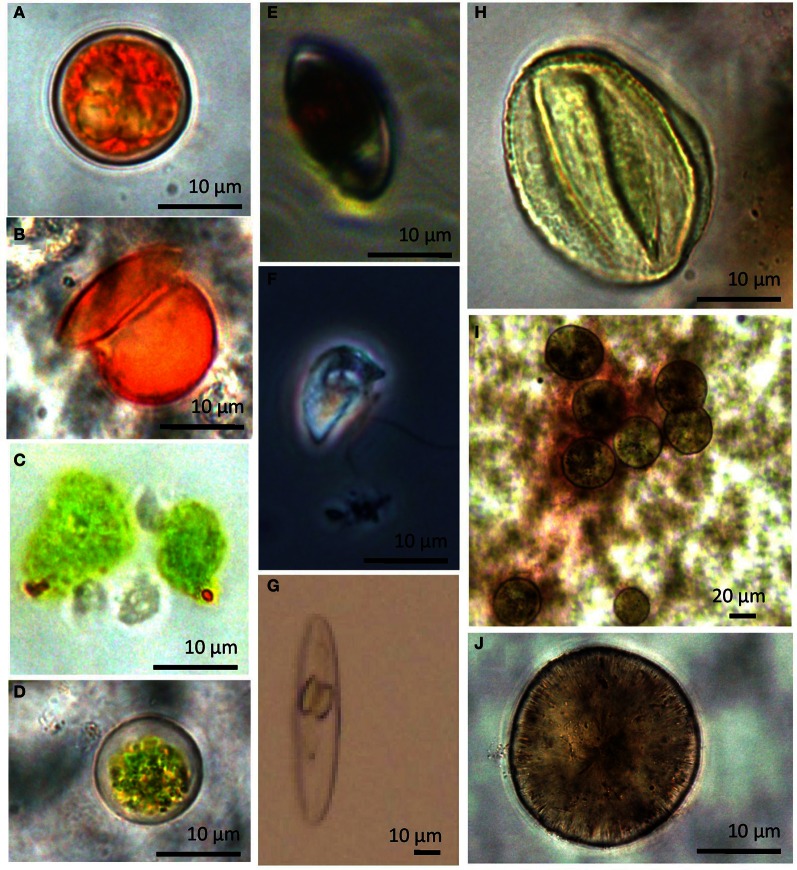
**Select light micrographs of lake protists collected in January 2010 when cells were exposed to high salinities and temperatures. (A)** Microscopic images showing *Dunaliella* cell aplanospores (cycts) and **(B)** an empty cell coat **(C,D)** Cells observed in cultures 12 months after collection **(E)** Heterotrophic flagellate, *Colpodella* sp. with ingested *Dunaliella* cell **(F)** *Colpodella* spp. **(G)** unidentified diatom observed in the halite crust sample. **(H)** unidentified ciliate from water sample. **(I,J)** unidentified diatoms from water samples.

The sole diatom (Bacillariophyta) sequence in our dataset fell within the *Navicula* spp. Diatoms like *Navicula* are commonly found but rarely abundant in hypersaline environments (Jarecki et al., 2006; Potter et al., 2006). We did note two additional diatom species in one of our crust cultures (Figures 10G,I, and J).

There was only one fungal clone in our dataset (LT85_J8), and it belonged to the phyla Basidiomycota. Given the low abundances, this group probably does not exert a significant contribution to the heterotrophy in the system. However, our PCR primer sets may not be ideally suited for characterizing fungal communities. Fungi commonly found in other hypersaline water are black yeasts and species of the Ascomycetes Eurotium and some Cladosporium and Basidiomycete species (Gunde-Cimerman et al., 2000; Butinar et al., 2005a,b; Zalar et al., 2005; Cantrell et al., 2006), but the understanding of their potential ecological role or input is still limited. Many fungal strains found in hypersaline samples also commonly occur in adjacent agricultural soils, which may suggest that fungal 18S rRNA signatures may be coming from land-based sources rather than local aquatic populations.

### Microscopic analysis of community composition in water samples

Microscopic analysis of water samples from the 2010 summer samples indicated an average abundance of 201 ± 121 SD cells ml^−1^ (85%) for *Colpodella* spp. and 32 ± 12 SD cells ml^−1^ (14%) for *Dunaliella* spp. Most cells of freshly collected *Dunaliella* were red, carotenoid-rich flagellated cell or unflagellated aplanospores (cysts) (Figure 10A,B). Samples collected in summer 2010 but maintained in cultures for 1 year at room temperature and lower salinities had a higher proportion of green cells (Figures 10C,D). All other groups combined made up ~1% (2 ± 1 SD cells ml^−1^) of the population. Counts were in fairly good agreement with clone library results.

## Discussion

Hypersaline systems are overwhelming dominated by prokaryotes, with communities dominated by the haloarchaea. However, eukaryotic organisms are also commonly present and play important ecological roles. Unlike prokaryotic communities, we did not see seasonal shifts in eukaryotic microbial communities corresponding to changes in physical parameters such as temperature and salinity. Summer 2007 prokaryotic abundances characterized microscopically averaged 1.6 × 10^7^ cells mL^−1^, with Haloarchaea making up 90% of the community and halophylic bacteria comprising 10% (Narasingarao et al., 2011). These were in agreement with community characterizations using genomic data. Summer communities averaged over multiple years were comprised of about 94% Haloarchaea (mostly *Haloquadratum, Halorubrum* and *Nanohaloarchaea*) and 6% halophilic bacteria (mostly *Salinibacter*). In winter the halophilic bacteria increased up to 32% of the community and overall diversity increased (Emerson et al., submitted). Interestingly, we found little seasonality in the microbial eukaryotic community structure despite wide temperature and salinity differences. This may suggest that grazing by heterotrophic flagellates in the system is more important than environmental factors in maintaining eukaryotic community structure.

Heterotrophic nanoflagellates are commonly found in hypersaline environments, and grazing has been shown to play important roles in other studies in re-mineralizing nutrients and in controlling prokaryotic and *Dunaliella* communities (Post et al., 1983; Patterson and Simpson, 1996; Park et al., 2003). For example, Park et al. estimated that a heterotrophic nanoflagellate in a Korean saltern could control prokaryotic communities with grazing rates high enough to turn over communities every 9–48 days. Intense grazing has also been shown to control *Dunaliella* populations in other hypersaline systems (Brock, 1975; Dolapsakis et al., 2005).

Colpodellids are small (12–19 μm), free-living predatory flagellates that are a sister group to the Apicomplexa (Kuvardina et al., 2002; Leander and Kuvardina, 2003). They have been identified in other hypersaline systems but historically have been considered a minor component in hypersaline environments (Guixa-Boixareu et al., 1996). However, in Lake Tyrrell, *Colpodella* dominated the microbial eukaryotic assemblage in the water for both clone libraries and microscope counts (Figure 3; Table 4). Cell densities in January 2010 samples were 201 cells mL^−1^ (9× greater than *Dunaliella* cells in the same samples). While the difference is greater when evaluating molecular sampling (31×), the general community composition reflected in the amplicon libraries was similar to microscopic enumerations. Differences could be attributed to differences in SSU rRNA gene copy number (Prokopowich et al., 2003; Medinger et al., 2010) or factors associated with 18S rRNA clone library construction biases (Koid et al., 2012). They have been observed to feed on a variety of other protists, but their ecology is generally not well understood (Post et al., 1983; Simpson and Patterson, 1996). Colpodellids generally feed by sucking prey contents partially or completely using a rostrum (Figure 10F) (Mylnikov, 2009), however those observed in this study readily ingested whole *Dunaliella* cells (Figure 10E).

Other heterotrophs were found in the lake for example (Figure 10H), although at much less frequency than *Colpodella*. Samples also had sequences that were closely related to *Trimyema koreanum*, a Plagiopylea ciliate first identified in a Korean saltern. Other ciliates were more closely related to *Fabrea salina*, a ciliate with remarkable tolerance to high salinities that has been routinely documented in hypersaline systems worldwide (Hauer and Rogerson, 2005) (Figure 8). Both *T. koreanum* and *F. salina* consume prokaryotes and have been shown to control populations of *Dunaliella* spp. (Post et al., 1983; Borowitzka et al., 1985; Dolapsakis et al., 2005; Cho et al., 2008). We propose that highly abundant co-occurring *Colpodella sp.* and other heterotrophs may exert considerable grazing pressure on communities of *Dunaliella* and prokaryotes.

*Dunaliella* have been described from a wide variety of habitats with salinities between 10% and saturation reviewed by Ben-Amotz et al. (2009) and Oren (2005). Densities have been reported as 1–3 × 10^4^ cells mL^−1^ in a salt saturated lagoon in Baja, Mexico (Olmos et al., 2009); 4–12.5 × 10^3^ cells mL^−1^ in a northern Greece solar saltern (Dolapsakis et al., 2005); and 4 × 10^4^ mL^−1^ during a surface water bloom in the Dead Sea, with an order of magnitude lower in deeper more saline water (Oren et al., 1995; Kaplan and Friedman, 2007). In relation to these studies, cell abundances in summer 2010 water samples were low (32 ± 12 SD cells ml^−1^). However, the sequence data showed a remarkably consistent summer and winter community composition in the water (Figure 3; Table 4), suggesting that differences in temperature and salinity may not be the main structuring factor controlling their abundances as has been suggested for other systems. Interestingly the sequence data of the halite crust indicated that *Dunaliella* dominated the community assemblage (Figure 3, Table 4). The crust may provide a refuge from predation and allow *Dunalliella* populations to dominate in this habitat.

Some species of *Dunaliella* over-produce carotenoids, especially β-carotene, and glycerol to balance the external osmotic pressure and to protect the chlorophyll and the cell DNA from the high irradiance from Ben-Amotz and Avron (1973), Avron and Ben-Amotz (1979), Oren et al. (1995), and Borowitzka (2007). Stressed cells appear orange-red rather than green. The majority of the observed cells from the 2010 summer samples were bright orange and round (Figure 10A). However after these sampled were maintained in laboratory cultures but transitioned to lower temperatures and salinities the *Dunaliella* in the cultures were mostly pear-shaped and green from chlorophyll pigments in their chloroplasts (Figures 10C,D). This suggests that either *Dunaliella* are quite physiologically plastic and when not stressed, and cells return to a more preferred growth state, or that the composition of the *Dunaliella* species community shifted in the altered environmental condition. The lake has a mixture of *Dunaliella* species (Figure 7). Some of our sequences align closely to *D. parva* and *D. viridis*, which are not known to readily alter their morphology and color at high salinities. Other sequences in our dataset align closer to other species (e.g., *D. salina*) that do assume an orange coloration and round shape when stressed (reviewed by Borowitzka, 1990). *Dunaliella* are considered potentially important in hypersaline systems as both primary producers and because the excess glycerol produced by some *Dunaliella* can be used as a substrate by Archaea (Elevi Bardavid et al., 2008). Future evaluations of the changes in *Dunaliella* community structure in different seasons or in different parts of the lake would be helpful in elucidating differences in ecological roles of the different species within the lake.

## Conclusion

The presence of microbial eukaryotes in hypersaline environments is not new. However, data on quantitative identification of communities of eukaryotic assemblages is limited. In addition to providing a phylogenetic survey of taxa based on near full-length 18S rRNA sequences, we found several novel groupings of organisms. Logares et al. (2006) suggested that relatively isolated systems would promote opportunities for rapid evolutionary divergence. It is unclear if these sequences are truly diverging from all other sequences or if the unique clustering of sequences is more of an artifact of a relatively small public database of characterized eukaryotic sequences with adequate length to reliably evaluate phylogenetic histories. This topic could be explored further with a more detailed focus on specific groups.

We also saw surprisingly minimal seasonal change in water column community structure, which may indicate that the system is controlled by top down grazers. However, the relative distribution of microbial eukaryotic communities living in the benthic halite crust was very different from water column communities. The crust may provide a refuge from predation for *Dunaliella* by the colpodellids and ciliates. The higher concentration of *Dunaliella* in the crust may also indicate that the crusts are an important source of autotrophic production. However, further work is needed to fully assess the role of primary production in the lake and the role that grazing has on the distribution of prokaryotes and autotrophic microbial eukaryotes (e.g., *Dunaliella*) within the system. These data provide the baseline information needed to further study the ecological context of the roles of microbial eukaryotes in the system.

### Conflict of interest statement

The authors declare that the research was conducted in the absence of any commercial or financial relationships that could be construed as a potential conflict of interest.
